# Epigenetic dysregulation and poorer prognosis in DAXX-deficient pancreatic neuroendocrine tumours

**DOI:** 10.1530/ERC-15-0108

**Published:** 2015-06

**Authors:** Christodoulos P Pipinikas, Harpreet Dibra, Anna Karpathakis, Andrew Feber, Marco Novelli, Dahmane Oukrif, Guiseppe Fusai, Roberto Valente, Martyn Caplin, Tim Meyer, Andrew Teschendorff, Christopher Bell, Tiffany J Morris, Paolo Salomoni, Tu-Vinh Luong, Brian Davidson, Stephan Beck, Christina Thirlwell

**Affiliations:** 1 Medical Genomics Laboratory, University College London Cancer Institute, University College London, 72 Huntley Street, London, WC1E 6BT, UK; 2 Department of Pathology, University College London, London, UK; 3 Department of Surgery, Royal Free Hospital, London, UK; 4 Royal Free Hospital NET Unit, London, UK; 5 CAS-MPG Partner Institute for Computational Biology, Shanghai Institute for Biological Sciences, Shanghai, 200031, China; 6 Department of Pathology, Royal Free Hospital, London, UK

## Dear Editor,

Exome sequencing of sporadic pancreatic neuroendocrine tumours (PNETs) has identified mutually exclusive mutations in the chromatin regulators α-thalassaemia/mental retardation X-linked *(ATRX) *and death-associated protein 6 *(DAXX)* genes in 43% of cases (18 and 23% of cases respectively in 68 cases studied) (Elsässer *et al*. 2011, Jiao *et al*. 2011). ATRX and DAXX are chromatin remodellers; their loss leads to alternative lengthening of telomeres (ALT) and chromosomal instability (CIN) (Heaphy *et al*. 2011). ALT is a telomerase-independent mechanism for the maintenance of telomere stabilisation. Although it was initially reported that *ATRX/DAXX* mutant tumours had superior 10-year survival and outcome (Jiao *et al*. 2011), a recent larger study on 243 tumours demonstrated that ATRX and DAXX loss and associated ALT in PNETs correlates with CIN, advanced tumour stage, the development of metastases and poorer progression-free survival (PFS) and overall survival (OS) (Marinoni *et al*. 2014).

ATRX interacts with DNA methyltransferases 3A and 3L (DNMT3A/3L), known as ATRX-DNMT3A-DNMT3L (ADD) (Hashimoto *et al*. 2010). DNMT3A and its accessory protein, DNMT3L, contain a histone H3 lysine 4 (H3K4) methyl-interacting ADD domain which links DNA methylation with unmodified H3K4. This interaction is one of the three described protein domains that provide a functional link between DNA methylation and histone modification. These interactions are pivotal for maintaining accurate replication of histone methylation patterns in newly replicated chromatin and in the subsequent fidelity of gene expression. ATRX interacts directly with DAXX, which functions as a chaperone for the deposition of the histone variant H3.3 at repeat sequences across the genome, including CpG islands and telomeric, pericentric and ribosomal repeats (Clynes *et al*. 2013). DAXX is a highly specific histone chaperone that discriminates H3.3 from other H3 variants. Mutually exclusive mutations in *ATRX *and *DAXX *are also found in neurological tumours, including neuroblastomas, paediatric glioblastomas, oligodendrogliomas and medulloblastomas (Clynes *et al*. 2013). Notably, H3.3 is mutated in paediatric glioblastoma and bone tumours, and H3.3 mutations are often associated with changes in global DNA methylation (Salomoni 2013, Maze *et al*. 2014).

Because of the known interaction between ATRX and DNMT3A/3L and the interplay between ATRX and DAXX, it is likely that PNETs with a loss of these tumour suppressor genes would have different genome-wide DNA methylation patterns as compared to those tumours that retain this function.

In this study, we sought to determine the genome-wide DNA methylation and copy number aberration profiles in ATRX/DAXX-positive and ATRX/DAXX-negative PNETs using the Infinium 450K HumanMethylation BeadArray (Illumina Inc., San Diego, CA, USA).

Only ATRX/DAXX-positive tumours and tumours with a loss of either ATRX or DAXX were considered. Included in the study were 12 age-matched, normal control pancreatic tissue samples (endocrine and exocrine) because of the extreme rarity of isolated pancreatic islet cell samples. Cases that showed heterogeneous ATRX or DAXX loss were also excluded so as to avoid difficulties in drawing firm conclusions.

**Figure 1:**
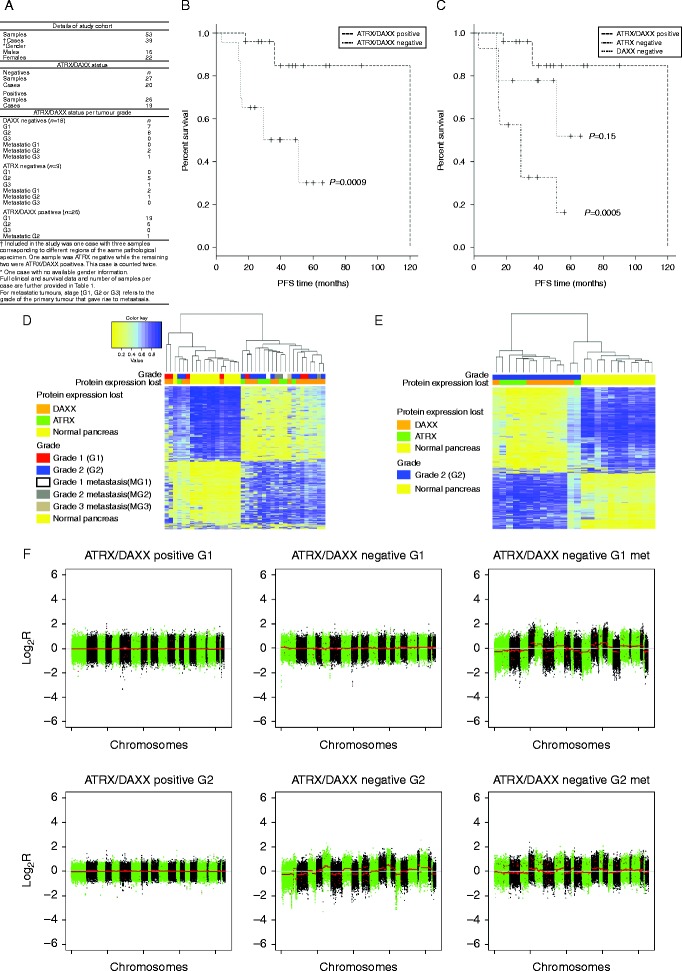
(A) Details of the study cohort. (B and C) Kaplan–Meier survival curves for ATRX- and DAXX-negative cases (*n*=17) as compared to ATRX/DAXX-positive cases (*n*=17) and ATRX-negative (*n*=8) and DAXX-negative (*n*=9) cases analysed independently and compared to ATRX/DAXX-positives cases respectively. The 5-year PFS was 85% for positive cases, 52% for ATRX-negative cases and 16% for DAXX-negative cases. (D) Unsupervised cluster analysis using the top 1000 MVPs that showed segregation of ATRX-negative (in green) and DAXX-negative (in orange) tumours as compared to normal pancreatic tissue. Of the six ATRX/DAXX-negative tumour samples (from six cases) that clustered with the normal control pancreatic samples, only one case (G1, DAXX-negative) progressed (PFS 51 months), and two cases had no follow-up data. Of the six metastatic samples included in this study, two of them (both DAXX-negative) had a matched G1 tumour sample. One of them grouped tightly with the G1 primary tumour. DNA methylation values (*β*-value 0–1) are represented using a colour scale, where yellow=low methylation and blue=high methylation. Samples are shown on the *x*-axis, and probes are shown on the *y*-axis. (E) Confirmation of tumour segregation primarily by grade and then by ATRX* or *DAXX status using the top 1000 MVPs. (F) Copy number variation (CNV) profiles associated with low and intermediate PNETs. The top panel demonstrates increasing CNV across the genome for low-grade (G1, ki-67 <2%) tumours, with the least CNV occurring in the ATRX/DAXX-positive primary (left). Increasing CNV alterations are seen in the ATRX/DAXX-negative tumour (middle) and most CNV alterations occur in the ATRX/DAXX-negative G1 liver metastasis (right). The same is observed in intermediate grade (G2, ki-67 3–20%) tumours in the bottom panel. Segmented copy numbers are shown as a red line, and sequential chromosomes are shown in green and black (chr. 1–22).

In total, 53 formalin-fixed paraffin-embedded tumour specimens (46 primaries and seven liver metastases from 39 cases) were included in this study (Fig. 1A). Of these, 27 specimens (51%) had lost either ATRX (*n*=9; 33%) or DAXX (*n*=18; 67%) protein expression, as determined by immunohistochemistry (Anti-ATRX (SAB4502258) and Anti-DAXX (HPA008736) antibodies (both rabbit polyclonal) were provided by Sigma–Aldrich). Endothelial cells that stained positive for ATRX and DAXX served as the internal control in each immunochemistry section from cases that lacked expression of the corresponding protein. Seven of 26 (27%) low-grade primary tumours (G1, ki-67 of <2%) exhibited ATRX/DAXX loss as compared to 13 of 19 (68%) intermediate-grade tumours (G2, ki-67 2–20%) (*P*=0.008, Fisher's exact test). One G3 and six of seven metastatic specimens also exhibited ATRX/DAXX loss.

Survival analysis was based on 34 cases with available follow-up data (eight ATRX-negative, nine DAXX-negative, and 17 ATRX/DAXX-positive cases; Table 1). Data were not available for two DAXX-negative and one ATRX/DAXX-positive case. Case 37 (counted twice), which had both ATRX-negative and ATRX/DAXX-positive samples, was also excluded. Analysis of the ATRX/DAXX-negative cases demonstrated that eight cases (five DAXX-negative and three ATRX-negative cases) progressed within 5 years of follow-up, whereas nine cases (four DAXX-negative and five ATRX-negative cases) did not. The majority of positive cases (13 of 17) remained progression-free over the period of study. Poorer 5-year PFS was observed in ATRX-negative and DAXX-negative cases as compared to ATRX/DAXX-positive cases (*P*=0.0009) (Fig. 1B). When ATRX-negative and DAXX-negative cases were analysed independently, a loss of DAXX led to a significantly poorer PFS at 5 years (DAXX loss: 16%, *P*=0.0005; ATRX loss: 52%, *P*=0.15; and no loss 85% 5-year PFS; Fig. 1C). All *P* values were obtained using a Cox regression model.

Depending on the different pair-wise comparisons, certain samples that had been assigned to unique arrays had to be removed to allow for the correction of the batch-to-batch variation which occurs when running the Illumina 450K HumMeth array. Comparison between all ATRX/DAXX-negative and ATRX/DAXX-positive tumours identified 58 methylation-variable positions (MVPs). Independent comparisons of either DAXX-negative (*n*=18) or ATRX-negative (*n*=7) with ATRX/DAXX-positive tumours (*n*=23) revealed 4352 MVPs and 34 differentially methylated regions (DMRs) and 258 MVPs and one DMR respectively. When ATRX-negative and DAXX-negative tumours were compared, we identified 196 195 MVPs and 6708 DMRs. Taken together, these observations demonstrate that genome-wide DNAm changes are associated with a loss of DAXX expression and not a loss of ATRX. A Benjamini–Hochberg (BH) adjusted *P* value of <0.05 that corrected for multiple testing (false discovery rate) was used throughout the various comparisons in order to identify significantly methylated variable positions.

When ATRX-negative and DAXX-negative tumours were compared as one group to normal pancreatic samples, we identified 133 938 MVPs (BH adjusted *P* value <0.05) and 4664 DMRs. A heat map that plotted the top 1000 MVPs for this comparison demonstrated that tumours were separated primarily by grade and then by ATRX or DAXX status (Fig. 1D). This was further confirmed by the unsupervised clustering of G2 tumours (eight DAXX-negative and five ATRX-negative cases) alone against normal pancreatic control samples for the top 1000 MVPs (Fig. 1E).

Of the 26 ATRX/DAXX-negative tumours (Fig. 1D), 20 samples (77%) were grouped into a distinct cluster. However, six ATRX/DAXX-negative tumour samples had intermediate methylation profiles and clustered with the normal control pancreatic samples (Fig. 1D). Of them, five were DAXX-negative (four G1 and one G2 tumours), and one was ATRX-negative (a G2 tumour). When comparing G2 tumours vs normal pancreas, there were 127 683 MVPs (adjusted *P* value <0.05) and 4337 DMRs. However, for G1 vs normal pancreas, 31 480 MVPs and 300 DMRs were identified. This indicates that low-grade (G1) tumours and normal pancreas have similar methylation profiles.

Copy number variation (CNV) was determined in ATRX/DAXX-negative and ATRX/DAXX-positive tumours using DNA methylation data as previously described (Feber *et al*. 2014). ATRX-negative and DAXX-negative tumours demonstrated increased CNV as compared to positive tumours (Fig. 1F). The genome-wide CNV rate was quantified by determining the cumulative size of genomic alterations (bp) in genomic regions that harboured a copy number change of <−0.3 (for loss) or >0.3 (for gain). Across the 23 ATRX/DAXX-positive primary tumours, there were 1.4×10^9^ bp of CNV in total (range per tumour 250 000–4.3×10^6^, median 1.6×10^6^). Across the 25 ATRX/DAXX-negative primary tumours, there were 2.4×10^9^ bp of CNV in total (range per tumour 279 000–8.0×10^7^, median 1.5×10^7^).

This is the first study of genome-wide DNA methylation in ATRX/DAXX-positive and ATRX/DAXX-negative PNETs. The finding of a higher incidence of ATRX and DAXX loss in intermediate-grade (G2) tumours may account for the worse PFS and OS previously observed (Marinoni *et al*. 2014); however, this finding requires validation in a separate clinical cohort.

Genome-wide DNA methylation analysis identified significantly more MVPs in DAXX-negative vs ATRX/DAXX-positive tumours as compared to ATRX-negatives vs ATRX/DAXX-positive tumours (4352 and 258 respectively). This suggests that DAXX deficiency drives genome-wide methylation changes, potentially through the functional loss of H3.3 deposition (which binds DAXX in a highly specific manner and loads it into DNA, whereas ATRX is a co-factor that is involved in DAXX targeting chromatin) and also through binding with the maintenance DNA methyl transferase *DNMT1 *(Salomoni 2013). Mutations in H3.3 occur commonly in paediatric brain tumours (Maze *et al*. 2014) and are known to lead genome-wide methylation changes. Because ATRX interacts with the *de novo *DNA methyl transferases DNMT3A/3L, its impact on genome-wide methylation in ATRX-deficient PNETs is less marked.

In addition to investigating genome-wide DNA methylation changes, CNV was determined from the non-normalised methylated and unmethylated signal intensities of the 450K array probes as previously described (Feber *et al*. 2014). In keeping with previously reported data in PNETs (Marinoni *et al*. 2014) and other solid tumours, we found a higher incidence of CNV alterations in tumours that had lost ATRX and DAXX (Heaphy *et al*. 2011). We also demonstrated that ATRX/DAXX-negative tumours have a poorer PFS as compared to ATRX/DAXX-positive cases (30 vs 85%, *P*=0.0009) and that DAXX-negative tumours have the poorest 5-year PFS overall (16%, *P*=0.0005).

We demonstrated that even though they have mutually exclusive mutations, ATRX- and DAXX-deficient PNETs have distinct genome-wide DNA methylation profiles. Loss of DAXX and not ATRX appears to be the driver event in altering genome-wide methylation changes in PNETs. Previous studies have analysed PNETs with ATRX and DAXX loss in a single cohort, because mutations in these genes are predominantly mutually exclusive. These findings are also relevant to other neurological tumours which are driven by ATRX and DAXX loss.

Finally, we demonstrated that DAXX-negative tumours have the poorest 5-year PFS, and we therefore suggest more aggressive disease course. However, further validation of these findings is warranted in a separate clinical cohort.

## Author contribution statement

H Dibra, M Caplin, T Meyer, P Salomoni, S Beck and C Thirlwell conceived and designed the study. C P Pipinikas, H Dibra, A Karpathakis, A Feber, M Novelli, D Oukrif, G Fusai, R Valente, A Teschendorff, C Bell, T J Morris, T-V Luong, B Davidson and C Thirlwell performed the data acquisition, analysis and interpretation. C P Pipinikas, H Dibra and C Thirlwell drafted the manuscript. C P Pipinikas, A Karpathakis, A Feber, M Novelli, M Caplin, T Meyer, A Teschendorff, C Bell, P Salomoni, S Beck and C Thirlwell critically revised the manuscript. C P Pipinikas, A Karpathakis, D Oukrif, T J Morris and T-V Luong provided technical support.

## Figures and Tables

**Table 1:** Clinical information and survival data

**Patient ID**	**Sample ID**	**Age**	**Gender**	**Primary/metastasis**	**Grade**	**Protein expression lost**	**Total follow-up (months)**	**PFS months**	**OS months**
**ATRX/DAXX-negative tumours**									
1	1B2T_MG2_N	46	M	Metastasis	MG2	ATRX	54	51	54
3	3A28T2_G3_N	53	M	Primary	G3	ATRX	83	14	83
4	4A4T_G2_N	60	M	Primary	G2	DAXX	85	3	85
7	7C3T_MG1_N	81	F	Metastasis	MG1	ATRX	60	No progression	Alive
8	8A2T_G1_N	49	F	Primary	G1	DAXX	NA	NA	NA
	8A3T1_G1_N				G1	DAXX			
	8A3T2_G1_N				G1	DAXX			
11	11A1T_MG3_N	56	M	Metastasis	MG3	DAXX	20	16	20
12	12A3T1_MG2_N	62	F	Metastasis	MG2	DAXX	106	29	Alive
	12A9T2_MG2_N			Metastasis	MG2	DAXX			
	12A10T_G2_N			Primary	G2	DAXX			
13	13A12T_G2_N	73	M	Primary	G2	DAXX	55	15	Alive
	13A13T_G2_N				G2	DAXX			
	13A14T_G2_N				G2	DAXX			
	13A19T_G2_N				G2	DAXX			
15	15A6T_G1_N	36	M	Primary	G1	DAXX	39	No progression	Alive
19	19A7T_G1_N	30	F	Primary	G1	DAXX	54	51	54
21	21A4T_G2_N	66	M	Primary	G2	ATRX	39	No progression	Alive
25	25A3T_G1_N	72	F	Primary	G1	DAXX	34	No progression	Alive
26	26A2T_G2_N	21	M	Primary	G2	DAXX	21	No progression	Alive
30	30C6T_G2_N	54	M	Primary	G2	ATRX	40	No progression	Alive
31	31A4T_G2_N	74	F	Primary	G2	ATRX	24	No progression	Alive
34	34A10T_G2_N	69	F	Primary	G2	DAXX	NA	NA	NA
35	35A7T_G1_N	23	F	Primary	G1	DAXX	56	No progression	Alive
36	36A3T_G2_N	57	M	Primary	G2	ATRX	72	14	72
37	37A3T1_G2_N	40	F	Primary	G2	ATRX	49	No progression	Alive
38	38A4T_MG1_N	84	F	Metastasis	MG1	ATRX	66	No progression	Alive
**ATRX/DAXX-positive tumours**									
2	2B2T_G1_P	67	M	Primary	G1	No Loss	90	No progression	Alive
5	5B5T2_G1_P	60	M	Primary	G1	No Loss	84	18	Alive
6	6A6T_G1_P	51	F	Primary	G1	No Loss	68	No progression	Alive
	6A8T1_G1_P				G1	No Loss			
	6A8T2_G1_P				G1	No Loss			
	6A8T3_G1_P				G1	No Loss			
9	9A9T2_G1_P	62	F	Primary	G1	No Loss	70	No progression	Alive
10	10A7T_G1_P	45	F	Primary	G1	No Loss	54	No progression	Alive
14	14B8T_G1_P	NA	NA	Primary	G1	No Loss	45	No progression	Alive
17	17A2T_G1_P	53	F	Primary	G1	No Loss	47	No progression	Alive
	17A4T_G1_P				G1	No Loss			
18	18A4T_G1_P	64	F	Primary	G1	No Loss	40	No progression	Alive
	18A5T_G1_P				G1	No Loss			
20	20A3T_MG2_P	69	F	Metastasis	MG2	No Loss	NA	NA	NA
22	22A6T_G1_P	72	M	Primary	G1	No Loss	18	No progression	Alive
23	23D2T_G1_P	54	F	Primary	G1	No Loss	28	No progression	Alive
	23D7T_G1_P				G1	No Loss			
24	24A6T2_G2_P	47	F	Primary	G2	No Loss	84	36	Alive
27	27A6T_G2_P	58	M	Primary	G2	No Loss	33	No progression	Alive
28	28A8T_G2_P	66	F	Primary	G2	No Loss	18	No progression	Alive
29	29C4T_G1_P	71	F	Primary	G1	No Loss	26	No progression	Alive
32	32B4T_G1_P	26	M	Primary	G1	No Loss	126	120	Alive
33	33E13T_G1_P	58	M	Primary	G1	No Loss	26	No progression	Alive
37	37A4T_G2_P	40	F	Primary	G2	No Loss	49	No progression	Alive
	37A5T_G2_P				G2	No Loss			
39	39IIFT_G2_P	58	F	Primary	G2	No Loss	162	36	Alive

PFS, progression-free survival; OS, overall survival; NA, not available. The three samples from case 37 are different regions of the same surgical pathological specimen. However, because of intra-tumoral heterogeneity, we observed that one sample was ATRX-negative, whereas the remaining two were ATRX/DAXX-positive. For this reason, case 37 was not considered in the survival analysis.
